# Expression of Fatty Acid Synthase Depends on NAC1 and Is Associated with Recurrent Ovarian Serous Carcinomas

**DOI:** 10.1155/2010/285191

**Published:** 2010-05-19

**Authors:** Stefanie M. Ueda, Kai Lee Yap, Ben Davidson, Yuan Tian, Vivek Murthy, Tian-Li Wang, Kala Visvanathan, Francis P. Kuhajda, Robert E. Bristow, Hui Zhang, Ie-Ming Shih

**Affiliations:** ^1^Department of Gynecology and Obstetrics, Johns Hopkins Medical Institutions, Baltimore, MD 21231, USA; ^2^Department of Oncology, Johns Hopkins Medical Institutions, Baltimore, MD 21231, USA; ^3^Department of Pathology, Johns Hopkins Medical Institutions, Baltimore, MD 21231, USA; ^4^Division of Pathology, Norwegian Radium Hospital, Rikshospitalet University Hospital, N-0310 Oslo, Norway; ^5^Faculty Division Radiumhospitalet, The Medical Faculty, University of Oslo, N-0310 Oslo, Norway

## Abstract

Our previous reports demonstrated that NAC1, a BTB/POZ domain-containing nuclear protein, upregulates in recurrent ovarian serous carcinoma and participates in developing drug resistance in cancer cells. The current study applies quantitative proteomics to identify the proteins controlled by NAC1 by comparing the proteomes of SKOV3 cells with and without expression of a dominant negative NAC1 construct, N130. From the proteins that are downregulated by N130 (upregulated by NAC1), we chose to further characterize fatty acid synthase (FASN). Similar to change in protein level, the FASN transcript level in SKOV3 cells was significantly reduced by N130 induction or by NAC1 knockdown. Immunohistochemistry showed that NAC1 and FASN immunointensities in ovarian serous carcinoma tissues had a highly significant correlation (*P* < .0001). Moreover, we found that recurrent serous carcinomas exhibited higher FASN immunointensities than their matched primary tumors (*P* < .001). Multivariate analysis showed that an FASN staining score of >1 in serous carcinomas was associated with a worse overall survival time (*P* < .01). Finally, C93, a new FASN inhibitor, induced massive apoptosis in carboplatin/paclitaxel resistant ovarian cancer cells. In conclusion, we show that NAC1 is essential for FASN expression in ovarian serous carcinomas and the expression of FASN significantly correlates with tumor recurrence and disease aggressiveness. The dependence of drug resistant tumor cells on FASN suggests a potential application of FASN-based therapeutics for recurrent ovarian cancer patients.

## 1. Introduction

Ovarian cancer is a neoplastic disease that exemplifies many of the major issues underlying current chemotherapy regimens in clinical oncology [1, 2]. Although most ovarian carcinomas at advanced stages are responsive to initial carboplatin and paclitaxel treatment, tumor clones resistant to these drugs eventually evolve as recurrent diseases. As a consequence, the main contributors to the mortality and morbidity of advanced stage ovarian cancer patients are chemoresistant tumors. In an effort to elucidate the molecular mechanisms underlying chemoresistance, we have studied ovarian cancer genome and transcriptome and have identified several genes and pathways that are potentially involved in this phenotype. One of these genes, *NACC1* encoding NAC1 (or NAC-1) protein, shows significantly higher expression in recurrent chemoresistant ovarian serous carcinomas than in primary untreated tumors [3]. *NACC1* belongs to the BTB/POZ domain gene family and contains the BEN domain that potentially mediates protein-DNA and protein-protein interactions during chromatin organization and transcription [4]. Biologically, NAC1 has been demonstrated to be an embryonic stem cell marker that controls proliferation and pluriopotency in embryonic stem cells [5–7]. NAC1 homodimerizes through the highly conserved BTB/POZ domain (a.a. from 1–129) [8] and its complex formation is essential for a variety of its biological functions. 

In our previous study, we found that recurrent ovarian serous carcinomas showed a higher NAC1 expression level than their matched primary untreated tumors [3, 9]. Ectopic expression of NAC1 increased paclitaxel resistance while knockdown of NAC1 or disruption of NAC1 homodimerization sensitized cancer cells to chemotherapeutic drugs [3, 9]. In order to understand how NAC1 contributes to drug resistance, we previously compared the gene expression profiles of SKOV3 ovarian cancer cells to those of NAC1 inactivated SKOV3 cells where the inactivation was induced by expression of N130, a mutant protein containing only the BTB/POZ domain of NAC1 that competitively inhibits NAC1 homodimerization. We found that NAC1 negatively regulated the components of the Gadd45 tumor suppressor pathway including Gadd45*α* and its binding protein, Gadd45gip1 [9, 10]. However, suppressing the Gadd45 tumor suppressor pathway did not completely rescue ovarian cancer cells after NAC1 inactivation, thus suggesting that other mechanisms also play a role. 

In this study, we identified proteins that are potentially regulated by NAC1 by applying a quantitative proteomic method using tandem mass spectrometry (MS/MS) and spectral count [11]. A total of 2914 proteins were identified. To reduce the sampling error and increase the quantification accuracy, 208 proteins identified by at least 20 spectra and two unique peptides were quantified to identify candidate proteins controlled by N130 expression. By comparing the protein quantity in the proteomes between N130-induced and noninduced SKOV3 cells, we identified new NAC1 regulated proteins that could be responsible for the NAC1-mediated drug resistance and for other biological functions. Among the proteins found to be downregulated in N130-induced cells, we selected fatty acid synthase (FASN) for further characterization because it has been shown to be associated with tumor progression in a variety of human cancers and its inhibitors are available for potential translation studies.

## 2. Methods

### 2.1. Cell Lines and Clinical Tissue Samples

High-grade ovarian carcinoma cell lines, SKOV3, A2780, and OVCAR3, were obtained from the American Type Culture Collection (Rockville, MD), and a low-grade serous cell line, MPSC1, was previously established by us [12]. OSE10 was as SV-40-immortalized ovarian surface epithelial cell line. All cell lines were maintained in RPMI media with 5% heat-inactivated fetal bovine serum (HyClone, Logan, UT) and 2% penicillin/streptomycin (Gibco, Rockville, MD). 

A total of 427 ovarian carcinoma tumors collected between January 1990 and December 2006 were arranged in tissue microarrays. These included 269 high-grade serous, 45 low-grade serous, 45 endometrioid, and 68 clear cell carcinomas. Fifteen histologically normal ovarian tissues and 20 benign ovarian serous cystadenomas collected over the same period were also analyzed. For Kaplan-Meier survival analysis, we included 184 primary high-grade serous carcinoma patients who underwent optimal primary cytoreductive surgery followed by platinum-based chemotherapy at the Johns Hopkins Hospital. The paraffin tissues were obtained from the surgical pathology repository at the Johns Hopkins Hospital and clinical data was obtained from medical records, including age, race, stage, histologic subtype, and date of patient's current status (alive or deceased). Median follow-up in those patients was 44.7 months. A subset of 56 high-grade serous carcinomas including 28 pairs of matched primary and recurrent tumors was analyzed to correlate FASN expression levels and to evaluate primary/recurrent tumor status. Another subset of 162 high-grade serous carcinomas was analyzed for correlation of NAC1 and FASN expression levels because the NAC1 immunostaining slides from these cases were available from our previous study [3]. Collection of tissue samples was in accordance with the guidelines of the institutional review board.

### 2.2. Quantitative Proteomics

Cell lysates were collected 48 hours after induction (removal of doxycyclin) or mock induction [3]. Protein concentration was measured by BCA assay. The same amounts of proteins (1 mg) from each condition were reduced, alkylated, and digested with 1 to 50 Trypsin/protein ratio at 37°C overnight. The peptides were purified with C18 columns and resuspended in water with a final concentration of 10 *μ*g/*μ*L.

For protein identification and quantification, each peptide mixture was analyzed twice by the LTQ ion trap mass spectrometer (Thermo Finnigan, San Jose, CA). For each analysis, 2 *μ*g of peptides were injected into a peptide cartridge packed with C18 resin, and then passed through a 10 cm × 75 *μ*m i.d. microcapillary HPLC (*μ*LC) column packed with C18 resin. A linear gradient of acetonitrile from 5%–32% over 100 minutes at flow rate of ~300 nL/min was applied. During the LC-MS mode, data was acquired in the *m/z* range of 400 and 2000. The MS/MS was also turned on to collect CID using data dependent mode. 

MS/MS spectra were searched with SEQUEST against the human IPI protein database (version 2.28). The peptide mass tolerance is 3.0 Da. Other parameters of database searching are as follows: cysteine modification (add cysteine 57) and oxidized methionine (add methionine with 16 Da). The output files were evaluated by INTERACT [13] and Peptide Prophet [14]. The identified peptides with a probability score ≥0.9 were used for the spectral count. To determine the number of MS/MS spectra used for identification of each protein in different conditions using our in-house developed software tool and to reduce the error for protein identification and quantification using MS/MS spectra, we only quantified proteins identified by at least 20 spectra and 2 independent peptides from the N130-induced and-noninduced cells.

### 2.3. Western Blot and Real Time PCR

Western blot analysis was performed on the protein lysates prepared from ovarian cancer cell lines and OSE10. Similar amounts of total protein from each lysate were separated on 10% Tris-Glycine-SDS polyacrylamide gels (Novex, San Diego, CA) and then electroblotted to Millipore Immobilon-P polyvinylidene difluoride membranes. The membranes were probed with an anti-FASN mouse monoclonal antibody (1 : 100) (FASgen, Baltimore, MD) followed by a peroxidase-conjugated goat antimouse immunoglobulin (1 : 6,000). Western blots were developed by chemiluminescence (Pierce, Rockford, IL) utilizing glyceraldehyde-3-phosphate dehydrogenase as the loading control. To determine the mRNA levels of FASN, we performed quantitative real-time PCR using a BioRad iCycler. Total RNA was isolated with the TRIzol method (Invitrogen) and cDNA was synthesized from 2 to 5 *μ*g total RNA. FASN primer sequences were 5′-CATCCAGATAGGCCTCATAGAC-3′ (forward) and 5′-CTCCATGAAGTAGGAGTGGAAG-3′ (reverse). The expression of FASN was normalized to that of human amyloid beta precursor protein based with threshold cycle numbers calculated from duplicate measurements. Mean fold expression differences were further normalized to those of ovarian surface epithelium, OSE10.

### 2.4. Immunohistochemistry

For immunohistochemistry, paraffin sections after deparaffinization were incubated with a primary antimouse FASN antibody at a dilution of 1 : 50 in a 4°C moist chamber overnight. Negative controls included benign serous cystadenomas and normal ovaries. Two independent observers scored the FASN immunoreactivity using a categorical scoring system from 0 (not detectable) to 3 (intense) with the mean score recorded from triplicates. Among the FASN stained cases, there were 162 that had been previously stained with an anti-NAC1 antibody [3, 15].

### 2.5. Cell Number and Apoptosis Detection

Cancer cells were seeded in 96-well plates at 2.5 × 10^3^ cells/well for 24 hours and then were incubated with 100 *μ*L of the FASN inhibitor, C93, at concentrations ranging from 2.5 *μ*g/mL to 50 *μ*g/mL for 48 hours. After incubation, the number of viable cells was measured by the CellTiter-Blue assay (Promega, Madison, WI). These numbers were plotted against FASN inhibitor concentrations, and the value of IC_50_ (i.e., the C93 concentration at which cell growth dropped to 50% of the control level) was estimated. To detect early apoptotic cells, we grew ovarian cancer cell lines in 6 well plates (5 × 10^5^ cells/well) and treated them with C93 at their IC_50_ and with DMSO of equal concentration. The early apoptotic cells were quantified utilizing the Annexin V-FITC detection kit (Biovision, Mountain View, CA) and annexin V-stained cells were determined by flow cytometry.

After treating cells with C93 or DMSO, cells were trypsinized, washed, and resuspended in a solution containing 0.6% NP-40, 3.7% formaldehyde, and 11 mg/mL Hoechst 33258 in a phosphate buffered solution. The stained cells were then quantified by the BD LSR cytometer (Becton Dickinson, Franklin Lakes, NJ). Cell cycle analysis was performed using the CellQuest software (Becton Dickinson) and cells in the subG_1_ phase that represent the late phase of apoptosis were also measured.

### 2.6. Lentivirus Production and Transduction

Two NAC1 targeting shRNA plasmids previously cloned into the pLKO.1 (Sigma) and the packaging lentivirus plasmids were cotransfected into 293FT cells using Lipofectamine 2000 (Invitrogen. The sequences of NAC1 targeting shRNAs were 5′-CCGGCCCAAGTGAGATTG  CACATTTCTCGAGAAATGTG  CAATCTCACTTGGGTTTTTTG-3′ (shRNA-A) and 5′-CCGG  CAAGTACTACTGCC  AGAACTTCTCGAG  AAGTTCTGGCAGTAGTACTTGTTTTTTG-3′ (shRNA-C). Five hours after incubation, the transfection reagents were replaced with 10% FBS RPMI culture media supplemented with bovine serum albumin. The virus-containing supernatant was collected, the mixture was centrifuged, and polybrene was added to a final concentration of 8 *μ*g/mL. Transduction was carried out by adding 0.25 mL of virus supernatant to the SKOV3 cells. A second transduction was performed 24 hours later using the same protocol. shRNA or control (vector only) virus transduced cells were enriched by adding 2 *μ*g/mL of puromycin. Following this second round of transduction, cells were collected at 48 and 72 hours for quantitative real-time PCR analysis of NAC1 and FASN mRNA expression levels.

## 3. Results

### 3.1. High-Throughput Quantitative Proteomics Identifies FASN as an NAC1-Regulated Protein

To determine the possible mechanism by which NAC1 promotes drug resistance, we used the quantitative proteomics to compare the proteomes of N130-induced and-noninduced SKOV3 ovarian cancer cells using tandem mass spectrometry (MS/MS) and spectral count [11]. We were able to identify proteins that corresponded to a total of 2914 proteins. To reduce the sampling error and increase the quantification accuracy, 208 proteins identified by at least 20 spectra and two unique peptides were quantified to identify candidate proteins controlled by N130 expression. Based on the protein expression ratio of N130 noninduced to N130 induced cells (N130 OFF versus N130 ON), we listed the 19 proteins whose abundances were at least increased 50% (OFF/ON ≥ 1.5, Table 1) and identified 18 proteins whose levels were at least increased 50% (ON/OFF ≥ 1.5) by N130 induction (Table 1). Cells with induced expression of N130 showed significantly higher levels NAC1 expression than N130-noninduced cells (ON/OFF = 76.5), and all the identified peptides from NAC1 matched the N130 (BTB/POZ) domain, indicating the robustness of N130 induction. Since our primary interest was to identify the proteins that are upregulated by NAC1 (i.e., downregulated by N130), we selected fatty acid synthase (FASN) for validation and characterization since its level in noninduced cells is 1.57 times that in induced cells. We made this choice because, more than other proteins in the list, FASN has been associated with tumor progression and because specific inhibitors of FASN are available for further studies. The levels of FASN are less abundant in N130 induced cells than in N130-noninduced cells, suggesting that NAC1 inactivation by N130 suppresses FASN protein expression in SKOV3 cells.

### 3.2. Validation of NAC1-Dependent FASN Expression

**Figure 1:**
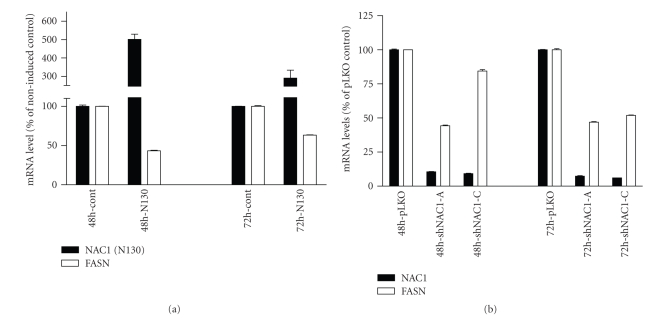
NAC1-dependent FASN expression in ovarian cancer cells. (a) N130 mRNA, as detected by a PCR primer pair that amplifies the N130 region, increases after N130 induction. In contrast, FASN mRNA decreases 48 and 72 hours after induction. (b) After shRNA lentivirus transduction, both shRNAs (-A and -C) effectively reduce NAC1 transcript levels to less than 10% of control and reduce FASN expression.

To validate the proteomic result above, we used quantitative real-time PCR to determine FASN transcript levels in two independent experimental systems. In our first system, we used the same NAC1 dominant negative (N130) cell model that was used in the proteomic analysis to assess whether disruption of NAC1 homodimerization by N130 leads to a decrease in FASN mRNA level. We observed that N130 mRNA, as detected by a PCR primer pair that amplifies the N130 region, increases 48 and 72 hours after induction (Figure 1(a)). At these same time points, SKOV3 cells with N130 induction exhibit a downregulation of FASN mRNA. Of note, at 48 hours after N130 induction, the decrease in FASN expression is similar between mRNA (46%) and protein (36%) levels. In our second system, we knocked down NAC1 in SKOV3 cells to determine the FASN levels. Two NAC1 shRNAs (shRNA-A and -C) that target different coding regions of *NACC1* were designed and packaged into lentivirus. We found that both shRNAs effectively reduce NAC1 transcript levels (to less than 10% of control) after transduction with shRNA lentivirus (Figure 1(b)). Correspondingly, FASN expression levels also decrease as compared to control. The above findings from cell culture systems suggest that NAC1 expression and its dimerization domain are essential for maintaining FASN expression in tumor cells. 

**Figure 2:**
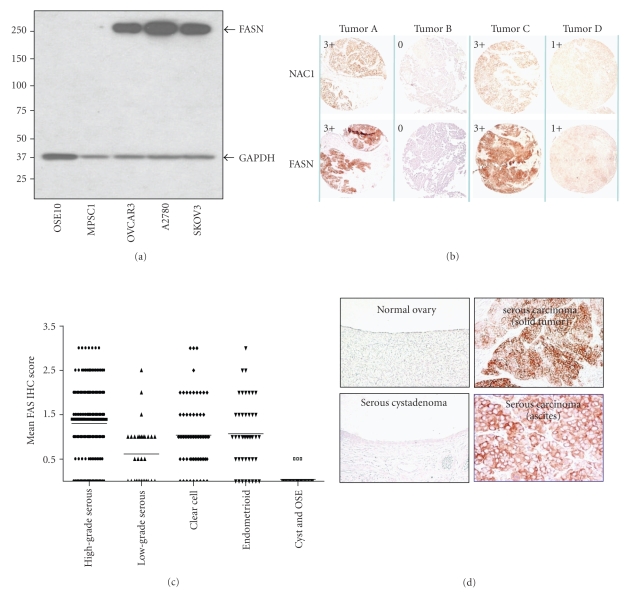
Expression of NAC1 and FASN in ovarian tumor tissues. (a) Western blot analysis shows a single protein band that corresponds to FASN protein in all three ovarian cancer cell lines (OVCAR3, A2780, and SKOV3) but not in a low-grade serous carcinoma cell line (MPSC1) nor in ovarian surface epithelial cells (OSE10). (b) Immunoreactivity of NAC1 (upper panels) and FASN (bottom panels) in four representative high-grade serous carcinomas. For each staining, the immunostaining scores are shown in the upper left corner (c)-(d). FASN expression in different types of ovarian carcinomas. (c) FASN immunointensity scores for different types of ovarian carcinoma including high-grade serous, low-grade serous, clear cell, and endometrioid carcinomas. The mean and standard deviation of the immunostaining score are shown. (d) Representative tumor sections of FASN immunostaining are illustrated. FASN immunoreactivity is detectable in high-grade serous carcinoma cells but not in normal ovarian surface epithelium nor in cystadenomas.

In order to assess the biological significance of NAC1-dependent FASN expression, we stained tumor samples obtained from ovarian cancer ascites and tissues for NAC1 and FASN and correlated their immunointensities. The specificity of the anti-NAC1 has been demonstrated previously [3] and the specificity of the anti-FASN monoclonal antibody is shown in Figure 2(a). Western blot analysis shows a single protein band at the molecular mass of FASN protein in all three ovarian cancer cell lines (OVCAR3, A2780, and SKOV3) but not in a low-grade serous carcinoma cell line, MPSC1, nor in ovarian surface epithelial cells (OSE10). Analyzing 162 high-grade serous carcinomas showed a significant positive correlation in NAC1 and FASN immunointensities (*P* < .0001, Fisher Exact test) (Table 2). These immunointensities for four representative high-grade serous carcinomas are shown in Figure 2(b). Our observations support the view that FASN expression is at least in part regulated by NAC1.

### 3.3. FASN Expression in Ovarian Serous Tumors

**Figure 3:**
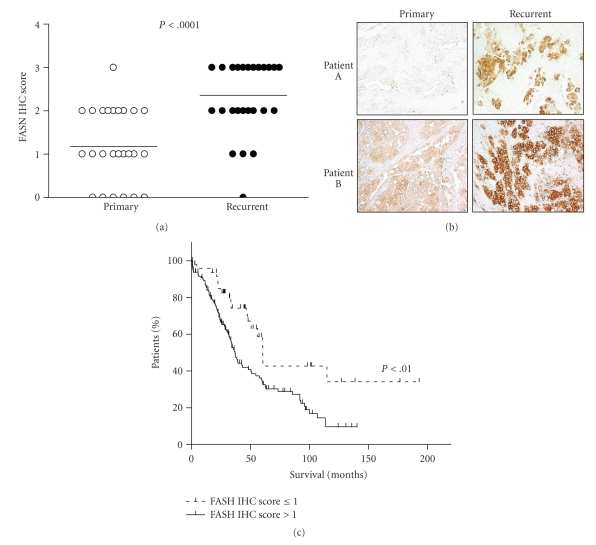
Clinical significance of FASN expression in high-grade ovarian serous carcinoma. (a) Comparison of FASN immunointensity scores for 28 pairs of matched primary and recurrent tumors from the same patients. Recurrent tumors exhibit elevated expression when compared to primary specimens from the same patient (*P* < .0001). (b) FASN staining of the primary and recurrent tumors from two representative patients. (c) Kaplan-Meier curve analysis shows that patients whose tumors exhibit higher FASN immunostaining scores have significantly shorter survival times than those whose tumors show undetectable or very low FASN immunoreactivity (60.4 versus 36.9 months, *P* < .01).

To extend the above immunostaining findings, we investigated FASN immunoreactivity in various types of ovarian tumors. All benign cystadenomas and normal ovarian surface epithelium (*n* = 35) display undetectable to very low FASN staining (mean  score = 0) whereas ovarian carcinomas of various histologic subtypes show FASN immunoreactivity (mean  score ≥ 1) in most cases (Figures 2(c) and 2(d)). The FASN staining score of high-grade serous carcinomas is significantly higher than that of low-grade serous carcinomas (*P* < .0001, *t*-test) and normal ovaries and benign cystadenomas (*P* < .0001). The FASN score in high-grade serous carcinomas is marginally higher than that in clear cell carcinoma (*P* = .024) and there is no statistical significance of FASN score between high-grade serous and endometrioid carcinoma (*P* = .099). Since NAC1 has been shown to be highly expressed in recurrent rather than primary high-grade ovarian serous carcinomas, we expect that FASN expression levels will follow the same pattern. To test this possibility, we assessed the FASN immunoreactivity of matched primary and first recurrent tumors from the same individuals. As expected, the FASN staining score is significantly higher in recurrent than in primary tumors (*P* < .0001, paired *t*-test) (Figure 3(a)). Based on a 2 × 2 contingency table and chi-square analysis, we also found that recurrent tumors exhibit a higher percentage of cases with intense FASN immunoreactivity than primary tumors do (Table 3). Figure 3(b) illustrates representative pairs of primary and recurrent tumors with FASN stain.

### 3.4. Clinical Significance of FASN Expression in High-Grade Ovarian Serous Carcinomas

To evaluate the clinical significance of FASN expression, we correlated the expression levels in primary high-grade serous carcinoma tumors with overall survival of the patient. We found that higher FASN staining scores (scores > 1) correlated with worse overall survival (*P* < .002) (Figure 3(c)). Patients with primary tumors that showed minimal or undetectable FASN immunoreactivity (score ≤ 1) had a median survival time of 60.4 months (range 1–193 months), whereas those with tumors that showed positive immunoreactivity (score > 1) had a median survival time of 36.9 months (range 1–140 months, *P* < .01). After adjusting for age, stage, and race in a multivariate analysis, we found that, in patients with primary serous carcinomas, FASN expression remains an independent marker for prognosis with a hazard ratio of 1.87 (95% CI: 1.12–3.11, *P* = .02).

### 3.5. C93 Suppresses Growth of Paclitaxel-Resistant and Carboplatin-Resistant Ovarian Cancer Cells

**Figure 4:**
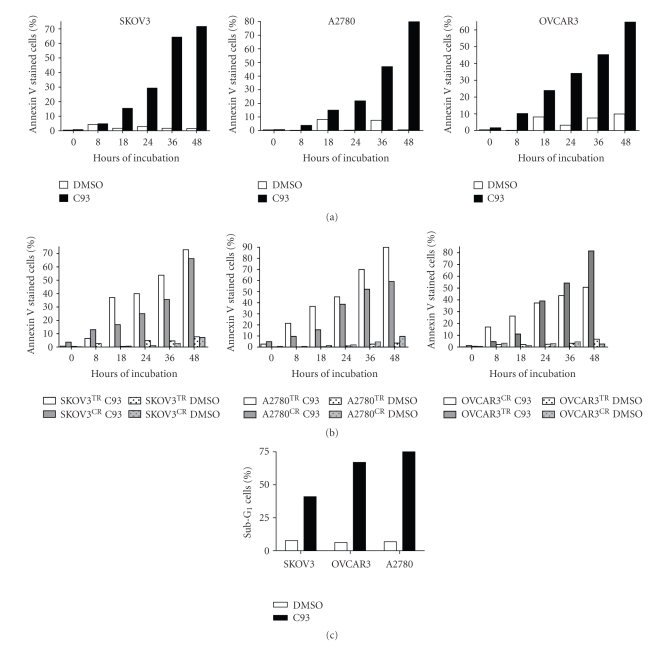
C93 treatment promotes apoptosis in ovarian cancer cells including cells that are resistant to carboplatin and paclitaxel. (a) In three ovarian cancer cell lines, C93 at its IC_50_ concentration increases the percentage of annexin V stained cells in a time dependent fashion. DMSO was used as the vehicle control. (b) C93 also increases annexin V stained cells in carboplatin resistant (CR) and paclitaxel resistant (TR) cell lines. (c) In all three cell lines, C93 treatment leads to an increase in sub-G_1_ cells when compared to the DMSO-treated group (*P* < .001). The percentage of sub-G_1_ cells is measured 48 hours after C93 treatment.

The above findings suggest that FASN preferentially expresses in recurrent and in most aggressive types of ovarian serous carcinomas, raising the possibility that FASN expression contributes to this phenotype. Thus, to determine if FASN expression is essential for cell growth and survival of high-grade serous carcinoma cells (including those that are resistant to paclitaxel or carboplatin), we applied C93, a second generation FASN inhibitor, to ovarian cancer cell lines (including SKOV3, A2780, and OVCAR3). We first used the cell number counted by CellTiter Blue assay to determine the IC_50_ of C93 for each ovarian cancer cell line. The IC_50_ of C93 was 7.4 *μ*g/mL, 7.4 *μ*g/mL, and 8.7 *μ*g/mL for parental, paclitaxel, and carboplatin resistant SKOV3 cells, respectively; 7.4 *μ*g/mL, 7.5 *μ*g/mL, and 8.4 *μ*g/mL for parental, paclitaxel, and carboplatin resistant A2780 cells, respectively; and 8.6 *μ*g/mL, 6.5 *μ*g/mL, and 8.8 *μ*g/mL for parental, paclitaxel, and carboplatin resistant OVCAR3 cells, respectively. When applied to each cell line at its IC_50_ concentration, C93 significantly increases the percentage of annexin V stained cells (representing the early phase of apoptosis) and the sub-G_1_ fraction in cell cycle analyses (representing the late phase) (Figure 4). Thus, C93 induces apoptosis in all three ovarian cancer cell lines. The number of annexin V-stained cells increases in a time dependent fashion in all cancer cell lines including those resistant to carboplatin and paclitaxel (Figure 4(b)). Moreover, paclitaxel resistant cells are more sensitive to C93 than carboplatin resistant cells, especially in the A2780 and SKOV3 cell lines. In all three cell lines, the C93-treated group shows a significantly higher percentage of sub-G_1_ cells than the DMSO-treated group (*P* < .01). These sub-G_1_ cells can be detected as early as 12 hours after C93 treatment and become more pronounced by 48 hours (Figure 4(c)). C93 fails to affect cell cycle progression in SKOV3 and OVCAR3 cells by 96 hours after treatment but it arrests A2780 cells in the G_1_ phase as early as 24 hours after treatment (data not shown).

## 4. Discussion

One of the major challenges facing ovarian cancer patients is the development of chemoresistant tumors after cytoreduction surgery and chemotherapy. In this study, we provide new evidence that homodimerization of NAC1, a drug resistance-associated nuclear protein, is essential for maintaining FASN expression at both protein and mRNA levels in ovarian cancer cells. We also demonstrate that FASN expression is highly correlated with the status of recurrent chemoresistant ovarian serous carcinomas and is independently correlated with poor overall survival. Suppressing FASN enzyme activity with its inhibitor induces apoptosis in cancer cells that are resistant to paclitaxel and carboplatin. Thus, molecular studies that illuminate the fundamental properties of chemoresistance should provide new therapeutic targets for treating recurrent ovarian cancers. 

Mammalian FASN is a ~260 kD cytoplasmic enzyme that is responsible for all the steps of *de novo* fatty acid synthesis. It catalyzes the NADPH-dependent condensation of malonyl-CoA and acetyl-CoA to palmitate [16]. Importantly, normal adult tissues express minimal amounts of FASN due to the presence of abundant dietary lipids. In contrast, a variety of tumors including breast, colon, ovary, and prostate cancer express elevated FASN levels [17–23], since tumor cells become less sensitive to regulatory nutritional signals and prefer the *de novo* lipogenesis pathway. Furthermore, FASN expression level highly correlates with the clinical aggressiveness of tumors [17, 18, 24–27].

Although previous reports have shown the role of FASN expression in ovarian cancer [18, 28], the current study provides new findings that should have several biological and clinical implications. First, we use in vitro cell culture studies using NAC1 targeting shRNAs and the dominant negative NAC1 (N130) approach to demonstrate that FASN is one of the proteins regulated at least in part by NAC1. This NAC1-dependent FASN expression in protein level is further supported by its mRNA level and the positive correlation between NAC1 and FASN immunointensities in ovarian serous carcinoma samples. The mechanism underlying upregulation of FASN in human cancer is not clear and it likely involves multiple pathways. Previous studies have shown that, in prostate cancer, caveolin-1 and KLF5/SREBP-1 function upstream of FASN [29, 30]. Thus, at least in ovarian cancer cells, the NAC1 pathway represents another mechanism for controlling FASN expression. Unlike other members of the BTB/POZ family, NAC1 lacks the zinc finger DNA-binding domain. Rather, it has been reported to act as a transcription corepressor with other BTB/POZ proteins [31]. Moreover, it has also been shown to interact with nuclear proteins potentially involved in tumorigenesis, including Nanog [7], CoREST [32], and HDAC3 and HDAC4 [33]. Thus, it is possible that FASN expression is indirectly controlled by NAC1 through binding with its specific partner(s). Identification of the NAC1-FASN pathway sheds new light on the molecular mechanism by which NAC1 promotes tumor progression. Further studies are required to elucidate the transcriptional regulation of FASN by NAC1. 

 The second finding in the current study involves the positive correlation between FASN expression and recurrence status in ovarian serous carcinoma tissue. It has been shown that ectopic overexpression of FASN results in drug resistance and that reducing the FASN expression increased the drug sensitivity in breast cancer cell lines [34]. At low concentrations, FASN inhibitor also sensitized tumor cells with FASN overexpression to chemotherapeutic agents [34, 35]. The FASN-mediated drug resistance appears to be due to a decrease in drug-induced apoptosis due to abundant palmitic acid as a result of FASN overexpression [34]. Despite recent advances in discovering drug-resistance biomarkers that are associated with recurrent and chemoresistant ovarian cancer [1, 3, 15, 36–41], reversing drug resistance by targeting these markers remains a tantalizing objective. The problem lies in the lack of reagents that can be used to inhibit these genes in clinical studies. Thus, therapeutic targeting of FASN provides an attractive option since selective small compound inhibitors for this protein are available. The first generation of FASN inhibitors, including C75 and Orlistat, potently inhibited tumor growth in a mouse xenograft model but the adverse effects associated with these drugs prevented their further consideration for clinical applications [23]. On the other hand, C93, the second generation FASN inhibitor used in this study, pharmacologically eliminates concomitant CPT-1 stimulation and does not induce the anorexia and feeding behavior changes in mice that were caused by earlier generation FASN inhibitors [28, 42]. These features are important for any FASN inhibitor before it can be considered for clinical testing. Our in vitro studies demonstrate that C93 affects both carboplatin and paclitaxel resistant ovarian cancer cells. Thus, ovarian cancer cells that overexpress FASN are molecularly dependent on it for cell survival. This observation is significant because FASN inhibitors provide an alternative treatment for ovarian cancer patients who have developed recurrent tumors after initial paclitaxel and carboplatin treatment. 

The antitumor effects of FASN inhibitors, like C93, are thought to result from depletion of end product fatty acids with accumulation of toxic intracellular malonyl-CoA and altered production of phospholipids with diminished membrane synthesis [23, 43]. Alternatively, FASN inhibitors may suppress tumors through metabolism-independent mechanisms. For example, FASN inhibition has been shown to selectively activate AMP-activated protein kinase (AMPK) in ovarian cancer cells causing cytotoxicity while sparing most normal human tissues from these pleiotropic effects of AMPK activation [28]. Moreover, a positive feedback regulation has been reported in ovarian carcinoma cells between AKT activation and FASN expression [44]. Phosphorylated AKT significantly correlates with FASN expression and FASN inhibition by either C75 or cerulenin downregulates phosphorylated AKT [44–46]. Therefore, FASN inhibitors may contribute to antioncogenesis by suppressing tumor-promoting signaling pathways such as AKT, a pathway that is frequently activated in ovarian serous carcinoma [47]. Thus, the C93-induced apoptosis in ovarian cancer cells may be related to FASN inactivation and/or suppression of AKT activity. It has been demonstrated that FASN inhibition initiates apoptosis more effectively in neoplastic cells with mutant *TP53* than in those with wild-type *TP53* [27, 48]. Our current finding supports this view as the A2780 cell line that harbors wild-type *TP53 * [49] responds to C93 with cell cycle arrest in the G_1_ phase in addition to apoptosis, while both the SKOV3 and OVCAR3 cell lines with their deleted and mutated *TP53*, respectively, respond to C93 with massive apoptosis [50]. Since mutated *TP53* appears in the majority of ovarian high-grade serous carcinomas [1, 51], it is likely that apoptosis is the predominant antitumor mechanism of FASN inhibition.

As with other epithelial cancers of the breast, colon, and prostate [20, 21, 52], FASN overexpression in ovarian cancer, as this study shows, appears to be associated with the most malignant type, that is, high-grade serous carcinoma. More importantly, in patients with this cancer, the level of FASN expression significantly correlates with worse clinical outcome, supporting the view that FASN expression contributes to disease aggressiveness in cancer cells. A similar observation has been reported for NAC1 [3, 15], hence suggesting that the NAC1-FASN pathway constitutes one of the mechanisms that propels ovarian cancer progression. How FASN contributes to disease aggressiveness in ovarian cancer remains speculative. Besides endowing drug resistance, FASN may enhance oncogenesis via cellular mechanisms such as enhancing the Wnt [53], c-Met [54], and proteosome pathways [55]. Moreover, upregulation of FASN gives cancer cells a growth and survival advantage by blocking apoptosis under hypoxia, a common condition in solid tumors and tumor effusions [56, 57]. 

In summary, this study identified candidate proteins controlled by NAC1 and provides new evidence that FASN expression is at least in part regulated by NAC1. Thus the NAC1-FASN pathway may represent a new mechanism for tumor progression that creates ovarian tumor cells that are resistant to chemotherapy. Our findings also indicate that FASN is a novel biomarker for recurrent ovarian serous carcinoma and its enzyme activity is essential for the survival of chemoresistant tumor cells. New generation FASN inhibitors, like C93, deserve consideration in future clinical trials involving advanced ovarian serous carcinomas, particularly those that are refractory to paclitaxel and platinum drugs. Further studies will be required to delineate the biological and translational roles of FASN in drug resistance in ovarian and perhaps other types of cancers. 

## Figures and Tables

**Table:** (a) Proteins downregulated by N130 induction

IPI	Protein name	ON-spectra	ON-peptides	OFF-spectra	OFF-peptides	Total spectra	Off/on
IPI00013881	Heterogeneous nuclear ribonucleoprotein H	4	5	20	7	24	4.62
IPI00012388	Transcription intermediary factor 1-beta	8	7	23	9	31	3.07
IPI00220985	Keratin, type I cytoskeletal 18	8	4	18	5	26	2.35
IPI00298547	RNA-binding protein regulatory subunit	10	7	23	7	33	2.30
IPI00299571	Protein disulfide isomerase A6	11	3	21	8	32	1.91
IPI00216976	aldolase C, fructose-bisphosphate	10	3	19	4	29	1.90
IPI00012074	Heterogeneous nuclear ribonucleoprotein R	9	5	17	7	26	1.89
IPI00013917	40S ribosomal protein S12	10	3	19	3	29	1.85
IPI00297779	T-complex protein 1, beta subunit	10	5	18	8	28	1.80
IPI00027434	Transforming protein RhoC	8	5	14	5	22	1.75
IPI00141318	P63 protein	8	6	13	5	21	1.73
IPI00219217	lactate dehydrogenase B	11	6	18	8	28	1.67
IPI00219096	high-mobility group box 1	15	4	25	5	40	1.67
IPI00009236	Caveolin-1	9	2	15	2	24	1.67
IPI00328343	Probable ATP-dependent RNA helicase p47	11	6	18	6	29	1.64
IPI00302927	T-complex protein 1, delta subunit	8	3	13	6	21	1.63
IPI00328188	fatty acid synthase	21	7	33	11	54	1.57
IPI00015027	AHNAK-related protein	11	7	17	11	27	1.55
IPI00017617	Probable RNA-dependent helicase p68	11	8	17	7	28	1.50

**Table:** (b) Proteins upregulated by N130 induction

IPI	Protein name	ON-spectra	ON-peptides	OFF-spectra	OFF-peptides	Total spectra	On/off
IPI00017454	Hypothetical protein FLJ13940	23	4	0	0	23	?
IPI00045207	NAC1 protein	153	9	2	1	155	76.50
IPI00395440	Unknown	26	5	5	4	31	5.13
IPI00107117	Peptidylprolyl isomerase B	14	5	6	3	20	2.33
IPI00290566	T-complex protein 1, alpha subunit	14	6	6	3	20	2.33
IPI00176692	similar to Heterogeneous nuclear ribonucleoprotein A1	27	5	12	6	39	2.25
IPI00221088	ribosomal protein S9	17	6	8	6	25	2.13
IPI00215918	ADP-ribosylation factor 4	17	7	9	7	26	1.89
IPI00015786	Spectrin alpha chain, brain	53	27	30	18	83	1.77
IPI00152412	Hypothetical protein	14	4	8	5	22	1.75
IPI00020984	Calnexin	29	10	17	7	46	1.71
IPI00010896	Chloride intracellular channel protein 1	17	5	10	4	27	1.70
IPI00027626	T-complex protein 1, zeta subunit	18	9	11	9	29	1.64
IPI00216587	40S ribosomal protein S8	13	6	8	6	21	1.63
IPI00216318	tyrosine 3-monooxygenase	40	10	25	9	65	1.58
IPI00329351	60 kDa heat shock protein, mitochondrial	66	21	44	17	110	1.52
IPI00217468	H1 histone family, member 5	21	5	14	4	35	1.50
IPI00291006	Malate dehydrogenase, mitochondrial	15	6	10	4	25	1.50

**Table 2:** Immunointensities of NAC1 and FASN in high-grade serous carcinoma.

	FASN 2+/3+	FASN 0/1+
NAC1 intense	51	34
NAC1 weak	19	58

Total (*n*)	70	92

**Table 3:** FASN immunointensity in primary and recurrent ovarian serous carcinomas.

	Score	
Tumor	0/1+	2+/3+
Primary	18	10
Recurrent	4	24

Total (*n*)	22	34

*χ* ^2^ *P* < .0001.

## Abbreviations

FASN: Fatty acid synthase.
NAC1: Nucleus accumbens associated 1.
